# The somatically preoccupied patient in primary care: use of attachment theory to strengthen physician-patient relationships

**DOI:** 10.1186/1750-4732-2-6

**Published:** 2008-04-29

**Authors:** Robert C Miller

**Affiliations:** 1 Assistant Professor, Neuropsychiatry and Behavioral Sciences Edward Via Virginia College of Osteopathic Medicine 2265 Kraft Drive Blacksburg, Virginia, 24060, USA

## Abstract

**Background:**

Individuals with somatic preoccupation constitute a substantial number of primary care patients. Somatically preoccupied patients are challenging to primary care physicians for several reasons including patient complaints consuming a great deal of physician time, expense to diagnose and treat and strain on the physician-patient relationship. This paper examines and discusses how disruptions in early attachment relationships such as often occurs when a female is a victim of child sexual abuse may result in somatic preoccupation in adulthood.

**Treatment utilizing attachment theory:**

Attachment theory provides a useful framework for primary care physicians to conceptualize somatic preoccupation. Utilization and containment techniques grounded in an understanding of attachment dynamics aid the physician in developing a sound physician-patient relationship. Successfully engaging the patient in treatment prevents misunderstandings that frequently derail medical care for somatically preoccupied patients.

## Review

The hallmarks of somatic preoccupation are that somatic symptoms do not rise to the severity level of or uniformly fit into the DSM-IV-TR diagnostic criteria characteristic of somatoform disorders. Somatic preoccupation is similar to somatoform disorders in that the patient has somatic symptoms which occur despite there being no causal disease or clinical finding [1]. Estimates of the prevalence of somatoform disorders in primary care range from 14% to as high as 26% [2-4]. Presumably, somatic preoccupation is also prevalent in primary care settings. Since a substantial number of patients in primary care settings meet DSM-IV-TR diagnostic criteria for somatoform disorders, it is likely that even more patients have sub-threshold symptoms characteristic of somatic preoccupation [5].

The prevalence of this condition in primary care points to the importance of developing empirically based conceptual models for treatment. Patients with somatoform health problems use twice as much inpatient and outpatient care and are twice as expensive to treat on an annual basis as are patients without a somatoform disorder [6]. Patients having actual somatization disorder are estimated to incur 6 to 14 times the U.S. average for health care expenses [7]. There is a substantial amount of medical literature world-wide indicating that, regardless of cultural background, somatically preoccupied patients pose a significant financial burden to health care delivery systems [8].

Health care systems are more and more conscious of providing efficient and cost-effective care; therefore, somatically preoccupied patients are a particularly challenging patient population to treat because conventional medical interventions useful in treating physical illness are not as successful with these patients. Somatically preoccupied patients constitute a large proportion of primary care visits [8] and it behooves physicians to be knowledgeable about the disorder and learn the necessary psychological intervention skills to work with it.

To ensure successful treatment of somatically preoccupied patients a high-quality physician-patient relationship must be established. It is well known that empathy has curative power [9,10], which is a particularly relevant aspect of treatment for these patients. A central proposition of this paper is that because osteopathic primary care physicians incorporate a holistic philosophy in their treatment methods they are well suited to treat somatically preoccupied patients. By understanding the underlying attachment dynamics of these patients, the osteopathic primary care physician can better comprehend and empathize with patients having this illness. The attachment theory model holds promise because it provides a method based in empirical study which can help doctors to understand *why *somatic preoccupation occurs. Based on this understanding physicians can utilize proven psychodynamic intervention strategies to promote patient health.

Part one of the paper conceptualizes somatic preoccupation through the attachment theory model. Three sections include:

• *Somatic Preoccupation and the Physician-Patient Relationship: *This section introduces how working with somatically preoccupied patients presents unique risks for physicians in terms of establishing and maintaining a good physician-patient relationship. Awareness of these potential risks lays the foundation for use of attachment theory through which the physician can effectively intervene and preserve the relationship with the patient.

• *Disrupted Attachment and Somatic Preoccupation*: This section examines how disruptions to healthy childhood attachment underlie adult somatic preoccupation and personality disorder. The section discusses the importance of physician awareness of the recapitulation of attachment dynamics that can arise in the physician-patient relationship.

• *Attachment Dynamics in the Physician-Patient Relationship – Somatic Preoccupation and Child Sexual Abuse History: *This section examines the connection between somatic preoccupation and patient history of child sexual abuse, and explores this connection in the context of the physician-patient relationship.

Part two of the paper, *Utilization and Containment Strategies for Treating Somatic Preoccupation*, provides specific strategies grounded in attachment theory, which can be useful to treating patients with somatic preoccupation. Use of techniques informed by the attachment model strengthens the physician-patient relationship and creates the potential for providing the patient a corrective emotional experience.

## Somatic preoccupation in primary care

### Somatic preoccupation and the physician-patient relationship

The primary care physician may be the *best *equipped health care professional to treat somatically preoccupied patients despite the predominantly psychological nature of the disorder. The primary care physician-patient relationship lends itself to establishing longer term and more interpersonally connected relationships with patients. The osteopathic primary care physician has a fuller understanding of the patient's health history, access to the patient's support network and a holistic appreciation of the interconnection of mind and body. Establishing honest and open communication in the relationship lends itself to helping somatically preoccupied patients come to terms with psychological and developmental aspects of their illness.

Somatically preoccupied patients are challenging to treat without effective psychological intervention tools. Patients may exhibit a confusing array of interpersonal behaviors and medical symptoms that place the physician-patient relationship at risk. Challenges facing physicians working with this patient group include making a differential diagnosis, establishing and maintaining rapport, coping with transference and countertransference reactions in the physician-patient relationship, managing patient demands on physician time, and engaging the patient in a viable treatment plan when the patient's willingness to participate is questionable. Ethical questions arise including when to "draw the line" in efforts to beneficently treat a patient who is unable to take advantage of the care offered, when to limit costly additional tests and procedures, and how to support the patient's autonomy when the patient is incapable of recognizing the etiology of their illness.

Somatoform disorders are chronic, persistent and abnormal personality traits are frequently present [11]. The disorders resist quick and simplistic explanations, and include psychological features about which the patient is largely unaware or uninterested. It is a formidable challenge for the doctor when the patient seeks a costly additional diagnostic procedure that in the physician's opinion may have little value to diagnosis or treatment [12]. More challenging still is the patient with a known disease who also has psychologically generated symptoms [13]. This patient may use the physical illness as proof that there are also other yet to be diagnosed illnesses. The patient, with this information in hand, pressures the physician to order more tests and procedures in hopes of uncovering the "real" physical problem for the unexplained illness.

The prognosis is poor for somatically preoccupied patients [1,11] in part because as treatment stalls, the frustration of patient and physician increases. The initial goodwill between patient and doctor wanes as treatment options diminish. It may be clear to the physician that the somatically preoccupied patient has a significant psychological component to their illness, yet it is common for the patient to have minimal insight about this as well as little interest in consulting with a mental health professional. The patient may challenge the doctor's professional competency if the patient perceives limited progress on resolving their problems. For example, patients who are told there may be a psychological cause to their medical problems can become defensive and argumentative. The patient may begin "doctor shopping" telling others that the former physician "told me it's all in my head." In fact, a central feature of somatic preoccupation is the patient's limited insight into psychological contributions to their medical problems.

The primary care physician who is hopeful that an early referral to psychiatry will resolve the patient's problems is therefore likely to be disappointed. The busy practitioner may be relieved when the patient moves on to another physician and may even unconsciously enable the patient to drop out of treatment so as to be rid of the emotional, physical and financial drain on the physician and the medical practice. As previously noted, however, patients with somatic preoccupation are frequent consumers of health care services. It is an occupational certainty that the primary care physician will see future patients with similar problems and be once again caught up in difficult physician-patient interpersonal dynamics. It is helpful for the physician to understand the etiology of these interpersonal dynamics so as to successfully prevent the recapitulation of the dynamics in future work with patients.

Inevitably the physician-patient interpersonal relationship moves front and center in the medical care of somatically preoccupied patients. Bass and Murphy [14] write that somatoform patients present with problems that make establishing a therapeutic relationship extremely difficult. They state that the "Breakdown of the physician-patient relationship is thus common, with the patient seeking medical attention elsewhere, often repeating the same pattern with doctor after doctor" [14, p. 404]. Early recognition aids in preventing the onset of serious challenges to the physician-patient relationship as well as setting the stage for more effective psychological interventions with the patient. The following section introduces Attachment Theory, which provides a model for recognition and intervention.

### Disrupted attachment and somatic preoccupation

Stable childhood attachment to adult caretakers is a core element of healthy life-span development. Sound attachment relationships foster a safe context for infants to explore the environment, build self-efficacy in the ability to function independently, and develop a foundation for mature adult relationships or object relations. Reliable early attachment relationships with adults serve to contain the child's anxieties and promote healthy separation-individuation in the developmental process. Conversely, when attachment relationships are unpredictable and unstable childhood anxieties increase and may ultimately result in adult interpersonal insecurities, an insecure sense of self, and displacement of anxiety into physical symptoms.

The childhood attachment process is therefore an essential and critical part of successful adaptation and growth. Early attachment bonds provide the foundation for later interpersonal and intrapersonal development [15,16]. Childhood attachment relationships create the schema from which individuals experience safety in relationships with others. Building on a sound attachment base the child can "maintain proximity to and contact with one or a few specific individuals who provide the subjective potential for *physical *and/or *psychological *safety and security" [17, p. 8] (emphasis added). In sum, healthy attachment formation underlies the child internalizing a sense of psychological and physical comfort. When healthy attachment is disrupted repercussions can be observed later in the individual's life [18-20]. Unstable or dysfunctional attachment relationships, for example, result in children developing avoidant or anxious/ambivalent relationship styles [15]. The anxious and avoidant child grows into adulthood insecure about self and ambivalent or even suspicious in relationships with others.

The predominant signs observed early in primary care treatment indicating the patient may have experienced dysfunctional early attachments are the patient's defensiveness about somatic symptoms and rigid interpersonal style. In fact, the fixed character style observed in patients with personality disorders is also observed with somatically preoccupied patients. Personality disorders and somatoform disorders are highly correlated with approximately two-thirds of the patients having a somatoform disorder also meeting criteria for a personality disorder [14].

Similarities are also likely to exist between somatically preoccupied patients and patients with personality disorders because both disorders are rooted in disrupted early attachment relationships. Somatic preoccupation and personality disorders share numerous commonalities including [14]:

1) Both disorders are persistent and enduring;

2) Problem behavior is typically present in adolescence and continues in personal and social adjustment in adulthood;

3) Both disorders have the characteristic feature of independence from mental disintegration (i.e. unlike schizophrenia, bipolar affective illness, or dementia);

4) Observed in cross-section, neither the somatoform or personality disorder patient will appear to be mentally disordered to an innocent observer. Yet to someone who knows the patient well over time a persistent problem will be evident.

5) Longitudinal studies of conduct disorder indicate that conduct disordered youth are prone to adult diagnoses of personality disorder and somatoform disorders.

6) Hypochondriacal patients are known to have prolonged duration of symptoms with poor long term adjustment, which is similar to personality disorders.

Psychological insecurity accompanies dysfunctional attachment for personality disorders and somatic preoccupation and leads to hypersensitivity to aches and pains, misattribution of the causes of physical symptoms, and exaggeration of symptom severity in an unconscious effort to receive comfort and reassurance. Insecurely attached patients are more anxious when they experience physiologic signals and in turn are more prone to misinterpret the signals as illness. As a result, the patient's anxiety spirals upward as does their help seeking behaviors. The patient will therefore persistently seek reassurance through frequent contact with their physician and maintain unrealistic hopes of finding a cure for perceived complaints and illnesses. From the point of view of the physician it may even paradoxically appear that for somatically preoccupied or personality disordered patients the more effort and service one provides the patient the more demanding and needy the patient becomes.

The physician's relationship with the somatically preoccupied patient will recapitulate the patient's early unfulfilling childhood attachment experiences. For physicians who have had experience with narcissistic or borderline personality disordered patients a similar quality to the nature of the relationship will be evident. Difficult patient transference issues arise in the physician-patient relationship and challenge the physician's ability to remain caring and empathic. For example, the patient may project unfulfilled dependency needs on the doctor, anticipate eventual rejection by the doctor (i.e. the relationship becoming a "self-fulfilling prophecy" wherein the patient seems to do everything possible to be turned away by the physician), and alternate between behaviors that idealize the doctor followed closely by angry and rejecting behaviors toward the doctor.

The physician who is unaware of these dynamics will unwittingly be susceptible to countertransference responses that fulfil the patient's life-script of rejection and hurt. The correlation between attachment problems and child sexual abuse history is instructive to helping physicians avoid this damaging recapitulation.

### Attachment dynamics in the physician-patient relationship: somatic preoccupation and child sexual abuse history

Child sexual abuse is linked to adult psychiatric disorders including major depression, borderline personality disorder, posttraumatic stress disorder and somatoform disorder [20-22]. Somatic symptoms commonly diagnosed among adult female survivors of child sexual abuse include chronic pain, pelvic pain, irritable bowel syndrome, fibromyalgia, chronic fatigue, facial pain, low back pain, gastrointestinal disorders, and migraine headaches [23-39]. Chronic pelvic pain alone is the top reason for 10% of outpatient gynecological consultations and 12–16% of hysterectomies in the United States [36,37].

The established prevalence of somatic preoccupation in primary care, the high incidence of somatic symptoms reported by adult female survivors of sexual abuse and the finding that child sexual abuse survivors use more health care than do non-abused women [38] establishes that primary care physicians will see numerous patients with a history of sexual trauma. Adult survivors of child sexual abuse may be challenging for physicians who do not recognize and anticipate the profound impact abuse history has on the patient's functioning, which includes an increased likelihood of somatic preoccupation.

Recognizing trauma in the patient's past alerts the physician to the potential that the physician-patient relationship will include special challenges and require the use of techniques specific to those challenges. In general, physicians learn that the presence of certain clinical phenomena is predictive of specific disease processes. Similarly, understanding that a history of trauma may underlie somatic preoccupation and attachment difficulties can create fruitful clinical hypotheses. For example, when the physician senses there is problematic patient transference (i.e. anger, skipped appointments, complaining to nurses or support staff) and recognizes one's own countertransference toward the patient (i.e. frustration, resentment and dread) the information can be useful if interpreted to understand the patient's illness rather than discounting it as a nuisance that adversely affects medical care.

The importance of reframing difficult patient interpersonal dynamics into useful clinical data is observed in research on sexual abuse history and malingering. In clinical settings actual genuine patient distress resulting from abuse trauma may appear to the health professional to be malingering [40]. If the physician perceives the patient to be deliberately exaggerating somatic symptoms then it is more likely the physician will also conclude the patient is doing so in hopes of achieving some secondary gain. Briere [41] and Briere & Elliot [42], however, describe that among child sexual abuse victims symptom exaggeration in fact reflects dissociation, posttraumatic stress disorder, depression, and poor family-of-origin background (i.e. disrupted attachment relationships). In other words, the consequences of a history of abuse will result in patients appearing to be exaggerating problems when in fact they are struggling with the psychological outcomes of a traumatic history.

The danger is that health care providers will minimize or discount legitimate patient problems if the provider is quick to assume exaggeration. Somatically preoccupied patients will have limited insight into the origins of their health problems, be unlikely to accept psychological interpretations for the problems, and have long-established psychological defenses to protect them from memories of the traumatic past. It is incumbent on the physician to appreciate and not overlook how trauma and attachment dynamics profoundly impact the medical care of the patient. By asking oneself, "Could this patient's apparent symptom exaggeration be connected with a history of abuse?" the physician can introduce into clinical hypothesis generation a potentially valuable source of clinical data. Furthermore, physician mindfulness about patient attachment history stimulates fruitful reflection on the meaning of challenging interpersonal dynamics in the physician-patient relationship.

Physician awareness can enhance rapport with the patient, stimulate the physician to creatively consider psychological contributions to the patient's presenting illness and lay the groundwork for a healing attachment experience in the physician-patient relationship. Reflection in this manner fosters the physician being more attuned to how a diverse range of childhood trauma experiences may potentially contribute to somatic preoccupation. Examples of other patient experiences that could result in attachment difficulties include a history of abandonment by parents, serious childhood illnesses or surgeries that resulted in prolonged hospital stays away from nurturing adults, and significant losses in childhood (i.e. death of a parent) that interrupted a positive attachment relationship.

Attachment theory is widely recognized as a valuable psychotherapeutic framework [43-50] and is demonstrated to be useful in treating somatoform disorders [14,18]. The first step to utilizing attachment theory is mindfulness that patient early life experiences impact and affect adult psychological and physical functioning. By achieving this awareness the primary care physician is uniquely positioned to capitalize on attachment dynamics because patient attachment dynamics will manifest in the relatively long term primary care relationship. For example, the primary care physician's role includes dimensions of caregiver and authority figure, which will naturally elicit underlying patient attachment dynamics. If effectively managed by the physician, attachment dynamics can be invaluable in fostering progress with the somatically preoccupied patient.

Thompson and Ciechanowski [50, p. 219] note the importance of physician awareness of attachment dynamics in stating, "Attachment theory offers a framework for physicians to *better understand and prepare for the clinical encounter" *(emphasis added). The authors emphasize the critical importance of the physician-patient relationship in effective medical care. The attachment perspective creates openings in the physician-patient relationship through which the relationship itself becomes a central element of healing. In sum, healthy attachment by the patient to the physician has the potential to become a corrective emotional experience for the patient. The corrective experience serves to counter problematic and longstanding intra-personal and inter-personal ways of being grounded in the history of trauma. In the next section tools are described to implement this corrective emotional experience with somatically preoccupied patients in primary care.

## Utilization and containment strategies for treating somatic preoccupation

Patient traumatic attachment experiences are recapitulated in medical care if the physician overlooks or misunderstands the power and influence inherent in the physician-patient relationship. Arnd-Caddigan [51, p. 296], for example, writes:

". . . subjects revealed that caregivers in childhood did not believe that the subjects were abused. The subjects had the parallel experience that physicians, counselors, and/or therapists did not believe that the subjects' somatic symptoms were real. Likewise, subjects had the experiences that caregivers held the subjects responsible for their abuse, and thought that the subjects behaved inappropriately in response to their past. The subjects also felt that doctors, counselors, and therapists, among others, told patients that they were responsible for their somatic symptoms and were inappropriate in their response to the symptoms."

Understanding attachment dynamics helps physicians attune to the needs of their patients and prevents the tragic recapitulation of mistrust and rejection in physician-patient interactions. *Utilization *and *Containment *are attachment therapy tools helpful to developing and sustaining good working relationships with somatically preoccupied patients. The goals of these strategies are to help patients take full advantage of primary health care services and to contain or prevent challenging interpersonal dynamics that arise in the physician-patient relationship and which can undermine effective patient care. Utilization and containment techniques promote patient emotional and physical healing and provide patients the opportunity to generalize this experiential learning to other interpersonal relationships.

The term *utilization *is chosen to emphasize that the physician *engages with the patient *so that challenging patient attachment behaviors such as neediness, help-rejection-complaining and blaming are reframed *as part of the illness *rather than perceived as an interference or disruption to treatment. Important as well is the physician's awareness and recognition that one's own emotional and behavioral reactions to the patient (i.e. frustration, dread, or withdrawal) must also be utilized. Utilization necessitates the physician recognize that attachment dynamics will *inevitably *recapitulate in the physician-patient relationship. Acknowledging this inevitability enables the physician to work with patient re-enactments of attachment dynamics rather than denying, avoiding or attempting to suppress them.

Whereas *utilization *connotes a collaborative interpersonal joining between doctor and patient, *containment *refers to the importance of boundaries in the relationship. Containment has the goal of "keeping in check" behaviors of both patient and physician that can threaten a productive physician-patient relationship. Whereas utilization *invites *attachment dynamics to occur in the relationship containment serves to *limit *the acting-out of dynamics that may threaten or derail treatment.

When applied together utilization and containment support the patient fully accessing healthcare resources while preventing destructive tendencies that undermine treatment. A paradox is inherent in the strategies because utilization accepts and even encourages attachment behaviors whereas containment reinforces limiting and placing boundaries on attachment behaviors. While this paradox seems counterintuitive it actually forms the basis for an emotionally healing relationship with the somatically preoccupied patient. The utilization-containment paradigm is synonymous with successful child rearing where the parent encourages attachment with the child while simultaneously supporting the child's need to separate and individuate. Similarly, the patient learns safety and trust in the relationship with the doctor while gaining confidence and self-efficacy to act autonomously without threat of punishment.

The significant power and influence inherent in the physician role will elicit both hope and fear for patients with a trauma history. By accepting and recognizing this significance the physician can *utilize *it in communications with the patient. Consider the following physician statements:

### *Physician statement 1*

"I want to help you but I can't help you unless you follow my instructions."

### *Physician statement 2*

"It's very important to me that my patients have input into their treatment. How can I help you feel included in the treatment plan?"

Statement 1 elicits guilt and shame for the somatically preoccupied patient because the statement clearly designates the physician as the predominant figure in the relationship. Beyond this obvious repercussion there is an important unspoken subtext in which the physician's statement means "I know better for you then you do." To a patient with a history of attachment trauma the subtext will feel similar to past relationships and be experienced as an infringement on safety and security in the relationship. The patient's past included powerful others forcing collusion in activities (i.e. sexual abuse) that violated trust and healthy attachment relationships. If the physician is unaware of this subtext there is the risk of unknowingly recapitulating trauma in the physician-patient relationship.

Statement 2 invites the patient to join with the physician in developing a treatment plan, validates the patient's ability to make good decisions, and offers help that can be accepted or rejected but in either case leaves open the possibility of additional discussion. In no way however does statement 2 undermine the physician's professionalism or expertise and good professional boundaries are maintained. Statement 2 strengthens the physician's role because it acknowledges the physician as the leader of the team who has the sound professional judgement to include patients in personal health care decision-making. But there is also an important subtext in statement 2 that is not verbally communicated to the patient. The subtext confirms that the patient is important to the physician, that the physician values having a trusting and collaborative relationship with the patient, and that the patient will not be forced into doing something for which they are uncomfortable and which would recapitulate trauma.

The example exemplifies how the use of language is critical to healing and that by effectively utilizing language, problems in relationships with patients can be reduced. If a physician pursues communications with a patient along the lines of Statement 1 it is foreseeable that the patient will resist recommendations and suggestions and be non-compliant in treatment. In the attachment paradigm non-compliance may actually be the patient's effort to be autonomous and self-efficacious. Physician authoritarian comment therefore become the source of patient resistance and non-compliance because the patient is determined to maintain an individuated self and not be controlled or abused.

Reframing problem behaviors into strivings for health enables constructive utilization of the patient's behavior rather than futile attempts to stop frustrating and aggravating non-compliance. Patient non-compliance is interpreted as the patient non-verbally stating, "you can't control or take advantage of me." Non-compliance therefore represents the patient's effort to meet legitimate needs for autonomy. Based on this conceptualization the central clinical question for the physician becomes, "How can I help the patient meet their needs in a way that isn't self-destructive?" rather then approaching non-compliance by asking "How can I make this patient comply with treatment?"

Despite a physician's best efforts to communicate effectively with somatically preoccupied patients disagreements and misunderstandings will inevitably occur. Containment strategies help the physician to predict, limit and manage potentially negative physician-patient interactions. Successful containment preserves the physician's interest and energy for long-term care of the patient, and prevents burn-out and potential emotional distancing from the patient. The core feature of effective containment is the use of empathic and validating responses to diffuse patient anger and rejection. Containment techniques "reality-check" the irrational beliefs and attitudes held by patients and offer new and healthier relationship experiences through immediate physician responses.

An effective containment technique is for the physician to periodically "check-in" with the patient about the quality of the physician-patient relationship. Physician inquiries about the relationship may at first feel unusual to the patient because in their past experience, particularly with health care providers, interpersonal sensitivity of this nature was rare. Similarly, the approach may feel awkward to the physician whose education may not have included process-oriented communication skills. With time the patient *and *doctor will likely come to respect and appreciate the opportunities to "check-in" on the quality of the physician-patient relationship because such opportunities serve to diffuse misunderstandings and miscommunications.

Effective use of containment by the physician appreciates that patient emotional outbursts stem from past dysfunctional interactions with powerful adults. If for example a patient screams at the doctor, "You never listen to me! You don't understand!" it is useful to recognize that emotion of this intensity is probably not completely grounded in the physician-patient relationship. One of the best means to address emotional negativity is for the physician to openly "name it" as a patient concern rooted in past relationships. For instance, acknowledging the patient's history of emotional pain with a statement such as "I can tell you are angry. I'm aware your relationships with physicians in the past have not always been helpful. I'd like to work with you to make our relationship different" draws attention to the fact the physician wants a good relationship with the patient and contrasts the current relationship with the patient's history of difficult relationships with physicians. The intervention is delivered in a measured and unemotional tone of voice to further diffuse and contain the patient's emotions by not meeting them with similar intensity. The response effectively models how this new relationship will regulate and manage strong affect. Strong patient emotions can become opportunities for teaching rather than something to be feared or avoided. Physician-patient interactions are utilized as elements of the healing process rather than framed as resistance or non-compliance.

If patient blaming and anger persists the physician must directly confront the behavior but this is not to imply the physician reacts out of personal frustration or anger. Responses along the lines of "You sound frustrated with me, help me better understand what that is about" contains anger by drawing attention to it and asking the patient for help in understanding the source of the unhappiness. The patient is invited to share more information so the physician can better understand. Disagreements between physicians and patients about medical care can unfortunately be a catalyst for larger problems and therefore require immediate and effective containment. Negative interactions left unattended and unresolved may eventually rupture the physician-patient relationship and result in complaints about the physician or even malpractice claims.

In the attachment theory paradigm the patient's history of relationships were marked by damaging and unresolved ruptures and the goal of the physician-patient relationship is to prevent such ruptures. In the past problems were ignored and "swept under the carpet" rather than acknowledged and worked-through. To prevent the recapitulation of problematic attachment dynamics the physician must make logical and reasonable statements to the patient such as "Let's talk about what just happened" and "I'd like to try to understand what just happened between you and me." Statements of this nature create openings for resolution rather than shutting down communication. Utilizing disruptions and containing harmful communications and behaviors allows the physician to demonstrate to the patient that the physician is caring and responsive, and models that relationships can be collaborative and non-abusive.

Despite their best efforts physicians will naturally from time to time have an empathic failure with a patient. If this occurs it is essential that the episode be immediately brought up for discussion with the patient. Patients having a history of damaged attachments will not generally acknowledge that they have hurt feelings. The patient will therefore not be the one to bring up the problem and is more likely to quietly dismiss the doctor, leave treatment and move on to another practitioner. The physician who is keenly attuned to attachment dynamics with somatically preoccupied patients will observe empathic failures as they occur and acknowledge and discuss the impact on the relationship.

Other common attachment recapitulations occurring in the physician-patient relationship with somatically preoccupied patients include neediness (i.e. frequent requests for additional appointments, testing or procedures), fearfulness (i.e resistance to referral to mental health) and, of course, preoccupation with health concerns (i.e numerous new health complaints and unexplained illnesses). If a patient complains that nothing seems to be helping relieve the symptoms a physician response such as "I can see that this is very frustrating and even exhausting for you. It must seem to you that as hard as you try nothing helps" acknowledges the patient's physical pain but perhaps more importantly empathically validates that the physician understands. Validating statements counter the patient's long held beliefs of isolation and insecurity by simply affirming to the patient, "I hear you and I understand."

Routine data collected in the medical practice can be valuable to increasing physician awareness that utilization and containment strategies would be helpful with a particular patient. For example, tracking outpatient visit data, observing referral patterns, attending to patient family history information, monitoring utilization, and being alert to co-occurring psychiatric conditions are relevant to identifying patient somatic preoccupation [5]. A family genogram, which can be quickly created in the presence of the patient, provides a point of reference for future discussions with the patient about their health history and their family history. If the genogram reveals that a parent of the patient was also somatically preoccupied the information can be helpful in understanding and treating the patient's symptoms.

In making inquires about family history of somatic preoccupation it is advisable to do so in a straightforward manner devoid judgment. The patient may be ashamed of their past and reluctant to openly discuss it. The direct approach creates a spirit of openness with the patient, reduces the shame patients feel about their past and family of origin, and models for the patient that it is acceptable to openly discuss problems with their doctor who in turn will be non-judgmental. Directness serves a number of containing functions including preserving the physician's energy for the patient and reducing the patient's anxiety and shame about past trauma.

Patients with depression and anxiety constitute up to 85% of the patients having somatic symptoms [4]. Stressful life events including marital problems or normative transitions such as a child leaving home can contribute to "flare ups" of somatic symptoms. If over time the primary care physician discusses with the patient how stressful events precede intensification of somatic complaints it will serve to contain or reduce the patient's anxiety and depression. For example, statements such as "We know in the past changes have resulted in you having more physical symptoms – that may be something to look for as you go through this change," can be reassuring for the patient.

Barsky [52] and Holder-Perkins et al. [53] recommend additional strategies that are helpful utilization and containment techniques. Physicians should focus on patient care rather than cure. This strategy has the effect of reducing patient emphasis on eliminating symptoms and reinforces coping skills. Conservative diagnostic and treatment approaches are recommended and careful review of records needs to occur before ordering new tests. The conservative approach prevents the patient from getting their hopes too high when tests are ordered as well as dropping too low when test results are inconclusive. Scheduling frequent and brief follow up appointments with the patient as well as sticking to the scheduled routine will be reassuring to the patient and serve to develop trust. Validate the patient's distress, do not refute symptoms and cautiously reassure the patient without giving false hope.

Frequent shifting between practitioners disrupts the patient being able to establish a healthy attachment to one provider and will increase the patient's anxiety. Consistent follow-up by one practitioner also allows for monitoring of substance abuse or self-medication with analgesics or benzodiazepines, for which somatically preoccupied patients are at risk [54]. Blackwell and DeMorgan [54] suggest linking presenting symptoms with the patient's day-to-day functioning, connecting symptoms with feelings by asking questions such as "How do you feel when you are having (the symptom)?," and obtaining the precise sequence of symptoms by exploring environmental triggers. These strategies invite a greater exchange of emotional information from the patient to the physician.

## Conclusion

Osteopathic physicians know that with the appropriate conditions the body has an amazing capacity to heal itself. The physician's awareness of attachment dynamics and attention to the physician-patient relationship through utilization and containment techniques compliment this holistic osteopathic philosophy of healing. The physician is better equipped to contemplate relationships with patients and use the relationship as an instrument of healing.

Platt and Gordon [55] refer to this contemplative process in medicine when discussing the frontispiece of Harvey Cushing's autobiography of William Osler. The frontispiece includes four views of Osler: palpating, auscultating, percussing, and finally *contemplating *the patient. It is in the act of contemplation that the physician steps away, literally and figuratively, to study the patient's condition as well as the physician's relationship to the patient.

The success or failure of primary care with somatically preoccupied patients resides in the quality of the physician-patient relationship. It is therefore useful for the physician from time to time to step away and assess the quality of the relationship. Many years ago the physician Andras Angyal [56, p. 318] proposed a set of questions that are as timely now as they were then. He asked, "Am I satisfied with the human aspect of our interaction? Are things all right between us and if not, why not?" Contemplative questions invite the somatically preoccupied patient into a partnership with the physician and model for the patient new and healthier attachments.

## Competing interests

The author declares that he has no competing interests.

## Author's contributions

RM was involved in conceptualizing this article, directed the development of the content, reviewed it critically, and provided final approval of the manuscript
